# Barriers and enablers associated with participation in a home-based pragmatic exercise snacking program in older adults delivered and monitored by Amazon Alexa: a qualitative study

**DOI:** 10.1007/s40520-022-02327-1

**Published:** 2023-01-17

**Authors:** Paul Jansons, Jackson J. Fyfe, Jack Dalla Via, Robin M. Daly, David Scott

**Affiliations:** 1 Institute for Physical Activity and Nutrition (IPAN), School of Exercise and Nutrition Sciences, Deakin University, Geelong, VIC Australia; 2 Department of Medicine, School of Clinical Sciences at Monash Health, Monash University, Clayton, VIC Australia; 3 Nutrition and Health Innovation Research Institute, School of Medical and Health Sciences, Edith Cowan University, Perth, WA Australia

**Keywords:** Exercise snacking, Exercise adherence, Barrier, Enablers, Telehealth, Older adults

## Abstract

**Background:**

‘Exercise snacking’, which is characterised by shorter and more frequent exercise bouts compared with traditional exercise guidelines, may be an acceptable strategy for increasing physical activity and reducing sedentary behaviour in older adults.

**Aim:**

The aim of this study was to evaluate the enablers and barriers for older adults associated with participation in a home-based exercise snacking program delivered and monitored using an Amazon Echo Show 5 device (Alexa).

**Methods:**

This study used an interpretive description qualitative design to conduct semi-structured interviews following a 12-week pilot study in 15 adults aged 60–89 years with at least one chronic condition. All participants were prescribed a home based, individualised, lower limb focussed ‘exercise snacking’ program (involving ≤ 10 min of bodyweight exercises 2–4 times per day) delivered and monitored by an Alexa. Qualitative interview data were analysed using thematic analysis.

**Results:**

All 15 participants (mean age 70.3 years) attended the semi-structured interview. Themes including time efficiency, flexibility, perceived health benefits, and motivation were enablers for participation in the ‘exercise snacking’ program. A lack of upper body exercises and omission of exercise equipment in the program, as well as a lack of time and motivation for performing exercise snacks three or more times per day, were barriers to participation.

**Conclusion:**

While ‘exercise snacking’ is acceptable for older adults, future trials should provide equipment (e.g. adjustable dumbbells, exercise bands), prescribe whole-body exercise programs, and establish strategies to support participation in more than three exercise snacks per day.

## Introduction

One-third of the Australian population have at least one chronic health condition, with the prevalence increasing to approximately 50% for those aged over 50 years [1]. Many older adults with chronic conditions report engaging in less than 30 min of physical activity per week, which has been associated with an increased risk of all-cause mortality over the subsequent three years [2].

To mitigate the burden of chronic health conditions, current best-practice guidelines recommend older adults undertake an individualised exercise program prescribed by a health professional [3]. However, adherence to a supervised exercise program delivered by a health professional has been reported to be less than 35% in community-dwelling older adults [4]. Key barriers to exercise participation in older adults include limited access to facilities and equipment, cost, a high perceived difficulty, and family and work responsibilities [5–7]. These factors highlight the need to identify more feasible strategies that limit barriers to exercise participation in older adults.

Emerging evidence suggests a home-based, brief (≤ 10 min) but frequent (e.g. 2–4 times per day) ‘exercise snacking’ program involving minimal-to-no equipment may be a feasible, safe and effective strategy for engaging older adults to participate in home-based exercise programs [8–10]. Exercise snacking trials have reported improvements in cardiorespiratory fitness [10] and/or markers of metabolic health [9]. There is also emerging evidence that exercise snacking may be feasible for enhancing muscle mass, strength and function [11–13]. One study in 20 older adults reported an adherence rate of 98% and improvements in lower body muscle (mean change 8 repetitions) in the 60-s sit-to-stand performance when undertaking a 4-week, twice-daily exercise snacking program, with no adverse events reported [11].

To our knowledge, only one qualitative study has explored the barriers and enablers to participating in an exercise snacking program [14]. Thirty-one healthy inactive adults aged 21–71 years reported that greater flexibility, convenience, and easier integration into activities of daily living were enablers to facilitate adherence in a five day only exercise snacking program. Furthermore, participants in that study reported that using digital health technologies to deliver and monitor an exercise snacking program with reminders would facilitate adherence [14]. Therefore, exercise snacking delivered and monitored using digital health technologies may be an acceptable strategy for engaging older adults to participate in home-based exercise programs.

We recently conducted the first trial to evaluate the feasibility of an individualised, home-based, asynchronously supervised exercise snacking program involving two to four daily exercise snacks in 15 community-dwelling older men and women aged 65–89 years with at least one chronic health condition. Our study reported an adherence rate of 115% to this 12-week exercise snacking program delivered and monitored via an Amazon Alexa Echo Show 5 (Alexa) by an accredited exercise physiologist, with no intervention-related adverse events reported [15]. The aim of this nested qualitative study was to explore the enablers and barriers of participating in a home-based, brief (≤ 10 min) but frequent (2–4 times per day) exercise snacking program after 12 weeks delivered and monitored by Alexa.

## Materials and methods

### Design

This qualitative study used an interpretive description qualitative design to conduct semi-structured interviews following a 12-week exercise snacking pilot study in 15 adults aged 60–89 years with at least one chronic condition. The period of recruitment for the 12-week pilot study was August 25th to September 3rd 2020 [15] with the last participant completing their 12-week semi-structured interview for this qualitative study on the 23rd December 2020. Qualitative interview data were analysed for all 15 participants that provided consent. We used the Consolidated Criteria for Reporting Qualitative Studies to report this study [16]. The study was approved by the Deakin University Human Research Ethics Committee (2020–166).

### Participants

We used purposive sampling to recruit 15 participants from our pilot study [15], who were previous participants of exercise trials at Deakin University who provided consent to be re-contacted for future trials, as described in detail previously [15]. Participants were eligible to participate in the study if they were aged 60–89, English-speaking, living alone, had access to a home Wi-Fi network, and were cognitively and physically able to safely complete a remotely monitored and delivered 12-week exercise snacking program. Further detail on the eligibility criteria, recruitment strategy and quantitative outcomes for this study are available in the previous article [15].

### Intervention

**Fig. 1:**
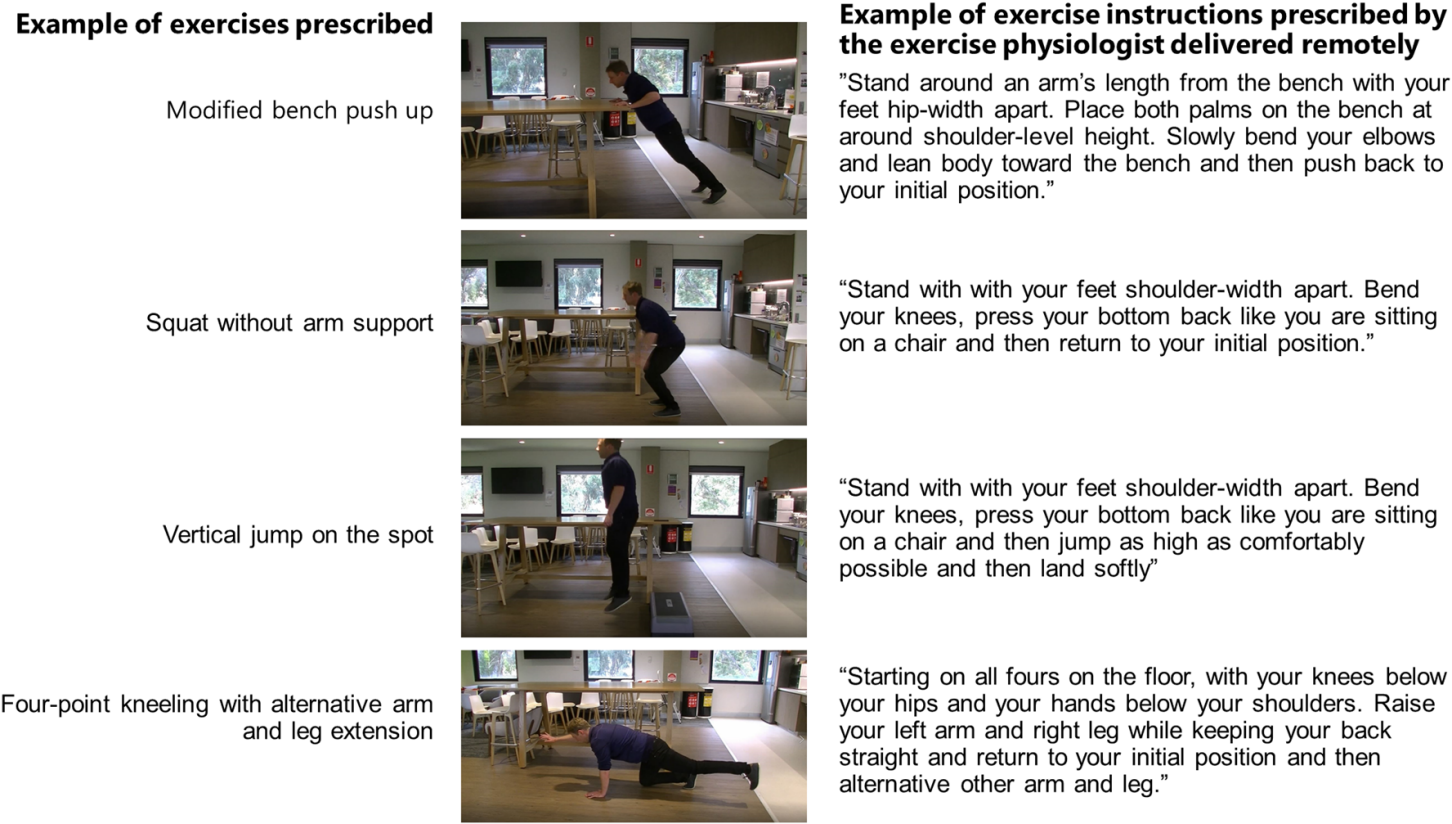
An example of an exercise snack delivered and monitored entirely remotely

All participants completed an initial consult with the accredited exercise physiologist via telephone to discuss exercise preferences/barriers and set a personalised exercise snacking program. The peak exercise professionals in Australia are referred to as ‘accredited exercise physiologists’. These professionals undertake a minimum 4-year university course and are qualified allied health professionals equipped with the knowledge, skills and competencies to design, deliver and evaluate safe and effective exercise interventions for people with acute, sub-acute or chronic medical conditions, injuries or disabilities. Participants were then prescribed an individualised, 12-week, home based, bodyweight only, brief and frequent (≤ 10 min of exercises progressing from two to four times per day over 12 weeks), remotely delivered and monitored exercise snacking program by an Alexa, as previously described [15]. The protocol initially delivered two ‘exercise snacks’ per day for the first four weeks of the intervention, three snacks per day for the second four weeks, and four snacks per day for the final four weeks. Each exercise snack was prescribed by an accredited exercise physiologist based on the *Osteo-cise: Strong Bones for Life* program [17]. Each snack consisted of four bodyweight exercises (e.g. modified bench push up, squat with no arm support, vertical jump on the spot and four point kneeling with alternative arm and leg extension) from a battery of exercises performed for 10-repetitions and with 30-s rest between each exercise. Each exercise snack was individually broadcasted using video demonstrations, and audio and written instructions via Alexa at specified times based on participant preference and distributed evenly throughout the day (see example of an individualised exercise snacking program in Fig. 1).

### Data collection

At follow-up, semi-structured interviews were conducted via videoconferencing by a study investigator (PJ) with expertise and experience in qualitative research [18, 19]. A set list of questions was designed to elicit responses to identify enablers and barriers of participating in the home-based exercise snacking intervention (S1). The study investigator asked further questions where necessary to clarify or obtain further information based on participant responses. All interviews were digitally voice recorded and were transcribed verbatim by a transcribing company (www.transcribeme.com TranscribeMe Inc).

### Data analysis

NVivo computer software (version 11, QSR International Pty Ltd, Doncaster, Victoria, Australia) was used to code the data and develop categories and themes. The six-stage process of thematic analysis as described by Braun & Clarke was used to analyse the data [20]. Data analysis was carried out by PJ (an accredited exercise physiologist who prescribed the exercise program) and JF with any disagreement resolved by DS.

## Results

Participants were 15 older adults, aged 65 to 79 years (mean age 70.3 years) with 60% (9/15) women. All participants reported at least one chronic condition, including hypercholesteraemia (53%, 8/15), hypertension (47%, 7/15), diabetes (40%, 6/15), osteoarthritis (40%, 6/15), any type of cancer (20%, 3/15), coronary heart disease (13%, 2/15), and asthma (13%, 2/15). Only one participant (6.7%) reported having a fall in the last 12 months.

**Fig. 2:**
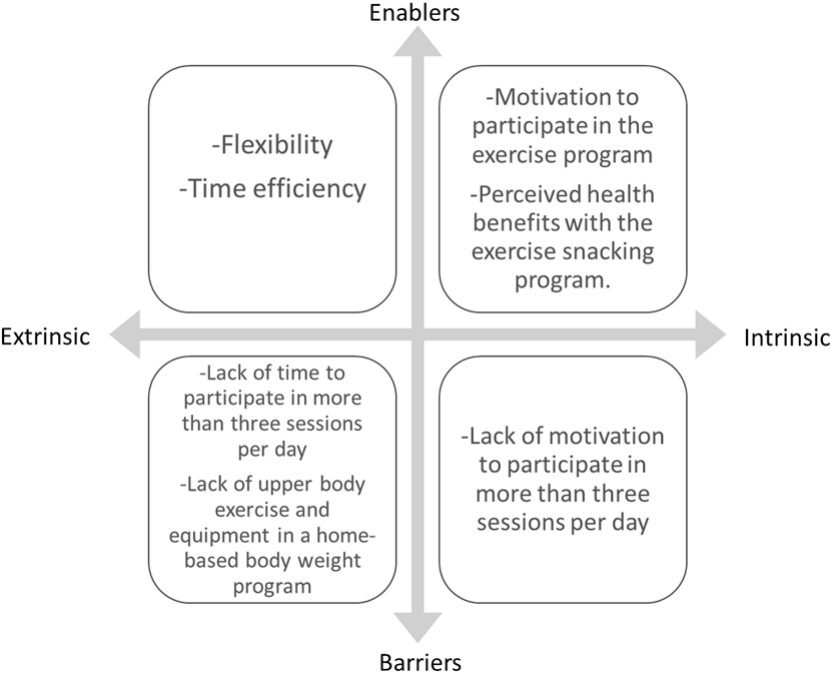
Enablers and barriers of participating in a home-based pragmatic exercise snacking program

Seven key themes were identified from the analysis of the semi-structured interviews, which are depicted in Fig. 2 as intrinsic and extrinsic enablers and barriers to participation in home-based exercise snacking. Themes including time efficiency, flexibility, perceived health benefits, and motivation were enablers for participation in exercise snacking. A lack of upper body exercise and omission of exercise equipment, and a lack of time and motivation for completing exercise snacks more than three times per day, were barriers to participation.

## Theme 1: Enablers of participating in an exercise snacking program

### Flexibility

Participants reported positive feelings towards the flexible nature of the ‘exercise snacking’ approach. Participants established a routine easily at home and integrated their exercise snacking program around their own activities of daily living. Some participants mentioned that the exercise snacks could be completed at any time of day, which allowed the exercise snacking approach to conveniently fit into their lives.

“The flexibility of a(n) [exercise] snacking was very good.” (Participant 8, female, age 67).

“If I wanted to do it four times a day–it doesn’t have to be two in the morning, two in the afternoon at a certain time. You pace at your own–there were times I did three lots, starting from seven in the morning until 10, and then the last one before I went to bed because I had to get out and do other things during the day. So you could pace it when you’re available.” (Participant 8, female, age 67).

### Time efficiency

Participants identified that it was time efficient to participate in the program since it involved only brief (≤ 10 min) exercise snacks. Some participants reported that distributing exercise into shorter but more frequent sessions each day was more acceptable than longer and less frequent exercise sessions, which are consistent with current exercise guidelines for older adults with chronic conditions [3].

“The snacking is a good concept, I think. It’s short and fast.” (Participant 15, female, age 74).

“I think it was a really, really good way to (get) you to do exercise because not everyone wants to get up and exercise for half an hour or an hour. And to be able to do it in little, short bursts I think is really beneficial.” (Participant 7, female, age 67).

### Motivation

The pragmatic nature of the exercise snacking program provided motivation to participate in the exercise program. Participants reported that the exercise snacking program allowed them to feel a sense of achievement because it was easy (or easier) to achieve.

“I did triple what you would expect, like instead of 10 I’d do 30 (repetitions). But I really think it was good having four different exercises each time.” (Participant 9, female, age 65).

“Yeah, because it’s do-able and there’s nothing stopping you from doing it more, or even repeating it if you wish to, or doing it faster.” (Participant 8, female, age 67).

“Yeah. So you’re in control. It just gave you the basic guidelines, which were not putting any pressure really, and if you wished to increase it, you could.” (Participant 8, female, age 67).

Some participants reported that gradually increasing the frequency of the exercise snacks (e.g. twice per day for the first four weeks of the intervention, three times per day for the second four weeks, and four times per day for the final four weeks) may facilitate greater adherence.

“The design of the exercise is excellent. I mean the first month I do two sets of exercises and the second month three sets and the fourth and then the third month, four sets of exercises. At the beginning when I did the two sets, I thought; oh, how will I be able to do the four sets? But when I came to the four sets, it wasn't difficult for me.” (Participant 10, male, age 66).

Having an accredited exercise physiologist prescribe the individualised exercise snacking program was perceived to improve adherence by contributing to accountability, exercise progression, and safety.

“The degree of the exercises was just, yeah it was doable. I was a bit afraid that you’ll (accredited exercise physiologist) make it too difficult and then I can’t do it anymore, there has to be challenge in it and it has to be just right, as Goldilocks says.” (Participant 3, female, age 67).

“I’m pleased that I had some exercises in a programmed and personal way to do, otherwise I’d just be sitting watching TV all day, or whatever. So that was very helpful.” (Participant 6, male, age 77).

### Perceived health benefits

Some participants reported desired and perceived positive health benefits associated with participation in the exercise snacking program, including improvements in pain, balance, flexibility, and strength.

“Okay. I found that I did become more flexible over time and I had a lot of problems with my knee, although there were some exercises I couldn't do because of it that were on the program but I did find that I felt that I became a lot more flexible and in general my knee became less painful.” (Participant 2, female, age 66).

“I’m hoping that I’ve built extra fitness over 12 weeks. Particularly balance, it’s not so much fitness, it’s balance that’s something that’s probably important for people in my demographic to be able to not fall over. And certainly, that balance with head turn is challenging. I need a finger that I can grab something if need be.” (Participant 15, female, age 74).

“I found out that my knees sort of work—well, not sort of, they work better now after the exercises.” (Participant 10, male, age 66).

Furthermore, some participants reported positive mental benefits from the ‘exercise snacking’ program, that included improvements in their emotional state.

“But overall it is—discipline helps to achieve what you would like to achieve and that’s what—I feel better and yeah, so I definitely felt physical exercise helped to sort you out emotionally, it stabilises emotions.” (Participant 3, female, age 67).

“The positive thing is well that you definitely realise after, I would say after six weeks I would say I realised that I get stronger. I have known my whole life, it enhances your mood if you do regular exercises.” (Participant 3, female, age 67).

## Theme 2: Barriers to participation in an exercise snacking intervention

### Lack of time

Some participants reported lack of time as an extrinsic barrier to participating in an ‘exercise snacking’ program, particularly when completing more than three ‘exercise snacks’ per day.

“The main time I enjoyed when it was—like, when I had to do it two or three times in the day. I found the fourth time, particularly at this point of time in the year, a bit challenging, really challenging. Not from a physical point of view but from a time point of view. Like, with Christmas, with Christmas shopping and Christmas—and the grandchildren dropping in, us driving down to the beach house to be with the rest of the family, it’s just like so difficult, the four sessions. I think personally for me I would find the reminders, two reminders a day really worked well and then half the time when it got to three or four—mainly four in the last four weeks I’d think, have I done it three times or have I done it four times?” (Participant 12, female age 68).

“We stuck to three exercises per day. When it got to four, it got a little bit time-critical, and because things have now opened-up post-pandemic, there are other things to do. I had to, not race through them, but perhaps do two lots in the morning, and then two lots in the afternoon to accomplish the four sessions. In other words, I wasn’t able to neatly space them throughout the day. There’s just too many other things going on. Sometimes I was just unable to do them at all, I was away visiting my daughter in the country. But on the whole, I think I did about 99% of them all, I did miss out on a few.” (Participant 6, male, age 77).

“Yeah, that starts to get a little bit onerous, only because just at this time of the year there’s just so many things that are happening. I got a little bit lost towards the end there, but I mean that was something that was my fault, nothing to do with the program as such I think. A different time, a different time of the year I think I’d be more comfortable with it.” (Participant 14, male, age 74).

**“**I couldn’t do four. I couldn’t fit in four. Hard enough doing two.” (Participant 13, female, age 66).

**“**Yeah. I found that two is enough because to try and fit it in and then to make sure that you fit it in, then it becomes taking over your schedule, your time, and then it’s not just—you want to get up and do some exercise and then do it later. Then it just sort of takes over and becomes inconvenient.” (Participant 13, female, age 66).

### Lack of motivation to participate in more than three sessions per day

Some participants reported a lack of motivation as an intrinsic barrier to participating in the ‘exercise snacking’ program, particularly when undertaking more than three sessions per day.

“Yeah, look, definitely four didn’t suit me. I struggled not so much physically, although it got boring. That was part of it. I just didn’t—my heart wasn’t in the [first] one, number one. Number two, I could never remember whether I had done three or four. And number three, it just was the wrong time of the year. So yeah, I didn’t like the last [lot]. I tried. I tried really hard but I didn’t like—wasn’t particularly engaged in that last—the [fourth] one.” (Participant 12, female age 68).

### Lack of upper body exercise and equipment in a home-based bodyweight program

Participants reported that the lack of both upper body exercises and equipment were barriers to participating in the ‘exercise snacking’ program, which was lower limb focussed and involved only bodyweight exercises.

“I didn’t think there were enough upper body exercises and there could have been a couple more they put in.” (Participant 5, female, age 79).

“I know the hand you’ve got push-ups, but you don’t have weights” (Participant 1, male, age 70).

“Now, I don’t have any exercise for the hands. Now, with the hand, as you know, we should have weights, anything—two bottles, two milk cartons for argument’s sake two kilo each hand, or the rubber stretching rubber exercise to whatever.” (Participant 1, male, age 70).

## Discussion

This nested qualitative analysis demonstrated that exercise snacking performed multiple times per day may be a feasible strategy for engaging older adults to participate in home-based exercise programs that are both delivered and monitored via an Alexa [15]. Participants reported themes relating to flexibility, time efficiency, and motivation as enablers for participation in the exercise snacking program. Conversely, participants reported some barriers associated with the ‘exercise snacking’ program, including the lack of upper body exercises, omission of exercise equipment, and a lack of time and motivation when exercise snacks were prescribed three or more times per day.

Our qualitative analyses revealed enablers related to flexibility and time efficiency associated with participation in the exercise snacking program. Some participants noted that distributing exercise into shorter but more frequent sessions each day was more acceptable than longer non-consecutive exercise sessions which are consistent with current exercise guidelines for older adults with chronic conditions [3]. These enablers were similar to those reported in the only other qualitative study exploring the barriers and enablers to participating in exercise snacking exercises. [14]. Participants in that study reported various enablers to participation in the exercise snacking program, including that it could be completed at any time of day and integrated into their own activities of daily living [14]. Another study evaluating the experiences in 38 community-dwelling older adults participating in an exercise snacking program revealed similar enablers related to flexibility and time efficiency [12]. The reason(s) for poor engagement with current exercise guidelines for older adults are multifactorial but include a high perceived difficulty and/or complexity, time constraints, limited access to facilities, and cost [21, 22]. It is possible that exercise snacking approaches could minimise these barriers for older adults. Future comparative qualitative studies are required to compare, and identify the determinants of, adherence to ‘exercise snacking’ program versus programs consistent with current exercise guidelines for older adults (e.g. American College of Sport Medicine) [3].

Participants in our study reported themes suggesting they favoured the brief but frequent nature of the exercise snacking intervention, which may have positively influenced their adherence to the program. It is possible that the shorter exercise snacks were perceived to be, and/or were, more achievable for this group of older adults. Participants in our study reported themes related to Bandura's social–cognitive theory of self-efficacy (i.e. people will choose to participate in a behaviour based on their expectation about their own ability to perform the behaviour) [23]. Therefore, participants in our study appeared to be willing to participate in the exercise snacking program based on self-efficacy that they would be able to complete the task prescribed. A cross-sectional study exploring self-efficacy in older adults found that greater levels of self-efficacy predicted participation in at least 20 min of continuous physical activity [24]. Furthermore, a qualitative study of 19 older men found those with a higher self-efficacy for participation in a supervised weekly exercise program had a greater ability to overcome barriers and prevent relapses [25]. Our main trial did not include a measure of self-efficacy associated with the exercise snacking program [15]; however it seems exercise snacks were perceived to be, and/or were, more achievable compared to other exercise prescriptions for older adults.

Some participants reported positive health benefits associated with the exercise snacking program, including improvements in pain, balance, flexibility, and strength, which were identified as enablers to their participation. These observations are consistent with the health beliefs model [26] whereby the perceived health benefits of an intervention predicts the likelihood of engaging in the associated intervention behaviours. A qualitative study involving sedentary older adults reported that perceived mental and physical health benefits provided motivation to complete 30 min of continuous moderate intensity physical activity on most days of the week [27]. However, the present study is the first we are aware of to demonstrate this finding in a brief (≤ 10 min) but frequent (2–4 times per day) exercise snacking program. Nevertheless, despite the pragmatic characteristics of the exercise snacking intervention, participants still perceived there to be positive benefits, and this may have contributed to the mean adherence (115%) to the exercise over 12 weeks [15].

Older adults in our study reported the lack of equipment used in the exercise snacking program as an extrinsic barrier to participation. The previous trial of home-based exercise snacking in 38 community-dwelling older adults similarly observed barriers related to the lack of additional exercise variety, equipment and upper body exercises [12]. These barriers were inconsistent to those reported in the only other qualitative study exploring the barriers and enablers to participating in exercise snacking exercises [14]. Participants in that study reported various enablers to participation in the exercise snacking program, including that it could be completed with no specialised equipment [14]. However, participants in our study may have felt after several weeks they felt familiar with, or insufficiently challenged by the body weight only 12-week exercise program. A qualitative study in older adults with chronic conditions found that home-based program participants reported that lack of access to exercise equipment, or not having the space to use their own exercise equipment, were associated with poorer adherence [19]. Gym participants in that study also reported that the equipment provided feedback to obtain further physical improvements (e.g. progressively increasing the resistance in a pin loaded knee extension machine), which may have also reinforced their motivation to continue to exercise [19]. Providing exercise equipment may provide a means to improve feedback, and support adherence and motivation in future home-based exercise snacking trials.

There is evidence to support our finding that some older adults report extrinsic barriers, such as a lack of time, to participation in home-based exercise. For example, a qualitative study involving sedentary older adults allocated to a home-based program, found that work and carer commitments were an environmental barrier to completing 30 min of moderate intensity physical activity on most days of the week [27]. A recent systematic review exploring barriers and facilitators to physical activity participation in older adults reported that in 40% of studies participants reported work and family responsibilities as barriers to recommended levels of physical activity [28]. Some participants in our study reported lack of motivation as an intrinsic barrier to participating in the exercise snacking program, particularly when exercise snacks were prescribed more than three times per day. Nevertheless, these barriers seemingly did not impact our participants’ mean adherence (115%) to the exercise intervention over 12 weeks. Some participants in our study reported themes related to self-determination theory [29] where they demonstrated the importance of autonomy to foster motivation and engagement with their prescribed exercise snacking program. It is possible that establishing individual preferences for participating in an exercise snacking program completed more than three times per day (e.g. performing two consecutive exercise snacks twice per day instead of evenly distributing four exercise snacks throughout the day) may alleviate some of these concerns in future exercise snacking trials.

Strengths of this study include the prescription of individualised and progressive exercise snacks delivered using a novel digital platform requiring minimal technology familiarity. Further longer term studies are required to determine the effectiveness and acceptability of exercise snacking programs in older adults when delivered and monitored remotely via different platforms, given it is likely that there is no single platform that meets the needs, preferences and technological literacy of all older adults; indeed, our previous study demonstrated that some participants would prefer a similar intervention delivered by platforms other than Alexa [30]. A limitation of this study may be selection bias because participants may have had positive feelings towards exercise trials given we recruited previous research participants who provided consent to be re-contacted for future trials. Data analysis was carried out by the accredited exercise physiologist who prescribed the exercise program which may have contributed to investigator bias. This study was conducted during the COVID-19 lockdowns/restrictions and our findings may not be representative of the experiences of participants in our trial outside this period of time.

## Conclusion

This qualitative study identified themes including time efficiency, flexibility, perceived health benefits and motivation as enablers for participation in the exercise snacking program. Future trials should consider providing exercise equipment, incorporate whole-body exercises, and establish participant preferences on achieving three or more exercise snacks per day.

## Data Availability

The data that support the findings of this study are available from the corresponding author upon reasonable request.
